# *Pythium oligandrum* Is a Type of Biocontrol Oomycete with Great Potential

**DOI:** 10.3390/jof12050375

**Published:** 2026-05-18

**Authors:** Kun Yang, Rongbo Wang, Liguang Liu, Kang An, Jitao Liu, Li Wang, Jianwei Shan, Chengchen Li, Liang Qi, Li Zheng, Xiaobo Li

**Affiliations:** 1 Guangdong Provincial Key Laboratory of Crop Genetic Improvement, Crops Research Institute, Guangdong Academy of Agricultural Sciences, Guangzhou 510640, China; ankang@gdaas.cn (K.A.); liu-j-t@163.com (J.L.); wangli20090617@163.com (L.W.); chanyujianwei@126.com (J.S.); lccazdb@163.com (C.L.); qiliang@gdaas.cn (L.Q.); 2 Fujian Key Laboratory for Monitoring and Integrated Management of Crop Pests, Institute of Plant Protection, Fujian Academy of Agricultural Sciences, Fuzhou 350013, China; wrb16128@163.com; 3 Key Laboratory of Green Prevention and Control on Fruits and Vegetables in South China, Ministry of Agriculture and Rural Affairs, College of Agriculture and Biology, Zhongkai University of Agriculture and Engineering, Guangzhou 510225, China; llgwx156@163.com (L.L.); bluestar183@163.com (L.Z.)

**Keywords:** eco-friendly control, non-pathogenic oomycetes, induced plant resistance

## Abstract

As a non-pathogenic oomycete, *Pythium oligandrum* possesses unique advantages, particularly in the context of being a biological control agent. With the increasing awareness of consumer consciousness, people are paying more attention to the use of environmentally friendly strategies in plant disease prevention and control. *Pythium oligandrum* is a type of biocontrol oomycete that can be developed as a biological control agent, and it does not have adverse effects on humans in the prevention and control of plant diseases. Consequently, there is increasing scientific interest in the beneficial plant–microbe interactions mediated by *P. oligandrum*. Currently, the main points of focus regarding the beneficial role of *P. oligandrum* in plant interactions are as follows: (i) *P. oligandrum* can activate plant defense responses and cause plants to produce resistance, thus protecting them from disease attacks; (ii) it is a strong mycoparasite that can coil around various oomycetes and fungi, directly killing pathogenic microorganisms; (iii) in addition, it can also promote plant growth. In this paper, we will discuss the aforementioned three main features in detail.

## 1. Introduction

Oomycetes are indeed microorganisms that can be found in diverse ecosystems, including tropical rainforests, oceans, deserts, and even extreme environments like Antarctica [1,2]. They have adapted to survive in a wide range of conditions and play important roles in these ecosystems. Currently, research on oomycetes has mostly focused on pathogenic species, while the study of non-pathogenic oomycetes such as *P. oligandrum* has been relatively limited. In 1930, *P. oligandrum* was first discovered in the root system of peas by Drechsler [2]. In 2020, Faure et al. reported the genome of *P. oligandrum* [3]. In soil, the interaction between *P. oligandrum* and plants is highly complex, and its exact role in these interactions has yet to be fully elucidated. *Pythium oligandrum*, as a biological control agent (BCA), not only activates plant defense responses but also kills pathogens through mycoparasitism, in addition to promoting plant growth. These effects make *P. oligandrum* an effective tool for plant protection, encouraging its wide use in agricultural production and ecosystem management.

## 2. An Efficacious Biological Control Agent for Boosting Plant Resistance Mechanisms

*Pythium oligandrum* is a biocontrol oomycete that can protect plants from pathogen invasion by inducing plant resistance. Through its symbiotic relationship with *P. oligandrum*, plants are better able to resist attacks from pathogens and improve their overall resistance levels. The mechanism of induced resistance plays a pivotal role in plant defense and protection. At present, *P. oligandrum* employs four primary mechanisms to induce plant resistance: (i) *P. oligandrum* and oospores trigger plant resistance: The presence of *P. oligandrum* and oospore suspension initiates a plant response, leading to the development of resistance against pathogens; (ii) *P. oligandrum* utilizes cell wall protein fraction (CWP) to induce plant resistance: The cell wall protein fraction released by *P. oligandrum* activate the plant defense response, bolstering their resistance against pathogens; (iii) *P. oligandrum* secretes microbe-associated molecular patterns (MAMPs) to induce plant resistance: The secretion of MAMPs by *P. oligandrum* triggers an immune response in plants, heightening their resistance against pathogens; (iv) *P. oligandrum* induces ferroptosis-like cell death to suppress pathogen infection: *P. oligandrum* triggers ferroptosis-like cell death as a defense strategy to protect plants from pathogen invasion. Considerable research endeavors have been directed towards investigating the above-mentioned four strategies, which serve as the primary means by which *P. oligandrum* achieves the goal of eliciting plant resistance.

### 2.1. Induction of Plant Resistance by P. oligandrum

#### 2.1.1. *P. oligandrum* Elicits Enhanced Resistance in Plants Against Pathogenic Oomycetes

*Pythium oligandrum* serves as an efficacious biological control agent through the elicitation of heightened plant resistance mechanisms. Multiple research studies have yielded extensive evidence demonstrating the efficacy of applying *P. oligandrum* in mitigating plant diseases induced by oomycetes, fungi, and bacteria (Table 1). Leveraging the use of *P. oligandrum* as a means to combat oomycete diseases proves efficacious in mitigating the detrimental effects inflicted by these pathogens. As early as 1997, Abdelzaher et al. reported the isolation of *P. oligandrum* from Egyptian soil, demonstrating its remarkable efficacy in controlling damping-off disease caused by *Pythium ultimum* on wheat [4]. In 1999, Ali-Shtayeh and Saleh reported that *P. oligandrum* was also effective in controlling diseases caused by *P. ultimum* on cucumber [5]. *Pythium oligandrum* has also been demonstrated to effectively control diseases caused by *Pythium aphanidermatum*. For example, the study conducted by Sayed et al. demonstrated that three strains of *P. oligandrum*, namely MS15, MS19, and MS31, were effective in controlling diseases caused by *P. aphanidermatum* on soybean [6]. The *P. oligandrum* strain D11 is also effective in controlling soybean damping off caused by *P. aphanidermatum* and *Pythium myriotylum* [7]. The strain GAQ1 of *P. oligandrum* is capable of significantly inhibiting Pythium soft rot of ginger caused by *P. myriotylum* [8,9]. Cultural filtrate of *P. oligandrum* (JU0328 and JU0329) significantly inhibited mycelial growth and the production of zoospores and oospores of *P. aphanidermatum* and *Pythium diclinum* [10]. Polyversum (*P. oligandrum*) is also employed for controlling *P. aphanidermatum* on tomato and eggplant [11]. Three strains of *P. oligandrum*, namely LMSA 1.01.631, CBS 109981 and CBS 530.74, have also been identified as effective agents in the control of diseases caused by *Pythium dissotocum* on tomato [12]. Furthermore, *P. oligandrum* induces significant resistance in two grapevine cultivars against *Plasmopara viticola* [13]. Together, *P. oligandrum* is currently being applied in the control of various pathogenic oomycetes, including *P. ultimum*, *P. aphanidermatum, P. myriotylum, P. diclinum*, *P. dissotocum* and *Pl. viticola*, in several plants including wheat, soybean, tomato, eggplant, and cucumber (Table 1).

#### 2.1.2. *P. oligandrum* Triggers an Elevation of Resistance Among Plants in the Battle Against Fungal Pathogens

*Pythium oligandrum* not only provides protection against oomycete-related diseases but also demonstrates efficacy in the control of fungal pathogens (Table 1). Of all the research conducted on *P. oligandrum*, it has been most extensively studied for its efficacy in the control of *Fusarium*. *Pythium oligandrum* has been utilized on wheat, barley and tomato to combat various *Fusarium* species, including *F. culmorum*, *F. oxysporum* and *F. graminearum* [14,15,16,17]. Yacoub et al. found that the *P. oligandrum* strains Po1, Po2, Po3 and Po37 could be applied for the control of both pathogenic species *Phaeomoniella chlamydospora* and *Neofusicoccum parvum* on grapevine [18,19,20]. Gerbore et al. discovered that *P. oligandrum* culture filtrate or Oligandrincan be used to effectively control *Erysiphe necator* on grapevine [21]. *Pythium oligandrum* applied to peppers can effectively control *Verticillium dahliae* and *V. Wilt* [22,23]. After treating sugar beet with *P. oligandrum*, it can effectively control seedling and taproot diseases caused by *Aphanomyces cochlioides* [24]. In addition, *P. oligandrum* can also prevent and control a variety of other fungal pathogens, including: *Sclerotinia sclerotiorum*, *Rhizoctonia solani*, *Botrytis cinerea*, *Sphaerotheca macularis* and *Mycosphaerella fragariae* [13,25,26,27,28,29] (Table 1).

#### 2.1.3. *P. oligandrum* Can Also Induce Resistance in Plants Against Bacterial and Root-Knot Nematode Diseases

We have already discussed that *P. oligandrum* plays a remarkable role in inducing plant defense mechanisms against pathogenic oomycetes and fungi. Furthermore, it exhibits the ability to stimulate plant defenses against bacterial pathogens as well. Implementing treatment of tomato plants with *P. oligandrum* demonstrates a significant potential to impede the onset of the bacterial pathogen, *Ralstonia solanacearum*, a causative agent of wilt disease [30,31,32,33]. Moreover, *P. oligandrum* is also used to control and prevent several root diseases. Pisarčik et al. found that Polyversum, also known as *P. oligandrum*, can effectively control root diseases in lucerne [34]. Polyversum 1 and Polyversum 3 are also effective in controlling root diseases in red clover [35]. *Pythium oligandrum* can also control root-knot nematode diseases on tomatoes [36]. *Pythium oligandrum* can also be used to prevent and treat some unknown pathogens in rapeseed and tomato [37,38]. Together, the use of *P. oligandrum* to induce plant resistance can prevent and treat various types of diseases, including those caused by oomycetes, fungi, bacteria, as well as pathogens that are yet to be identified (Table 1).

### 2.2. P. oligandrum Produces Numerous Molecules to Induce Plant Resistance

Pathogens secrete several substances that allow them to colonize and utilize the target host. Some of these substances, such as microbe-associated molecular patterns (MAMPs), are recognized by plants and initiate defense reactions [39]. *Pythium oligandrum* also produces an extensive array of molecules that exert influence on plants; these include Oligandrin, CWPs, MAMPs and RXLR effectors. These molecules can induce plant defense responses to varying degrees.

#### 2.2.1. *Oligandrin*, a Molecule Produced by the *P. oligandrum* Induces Resistance to Pathogens

A low-molecular protein, termed as Oligandrin, was meticulously extracted in its pure form from the cultural filtrate of *P. oligandrum*. Upon application to tomato plants, this protein exhibited the capacity to instigate plant defense mechanisms, thereby playing a pivotal role in confining the invasion of stem cells by the deleterious oomycete, *Ph. parasitica* [40]. Oligandrin demonstrates tangible effectiveness in instigating plant defenses against the onslaught of fungal pathogens. The application of Oligandrin triggered the expression of various defense-associated transcripts and, via diverse mechanisms, amplified resistance against powdery mildew in an otherwise susceptible tomato genotype [41]. Oligandrin also possesses the capacity to manage gray mold, and it may hold significant influence on the stimulation of resistance against *B. cinerea* [42,43,44]. Oligandrin initiates a systemic resistance to Fusarium crown and root rot within tomato plants [45]. Moreover, Oligandrin enhances tomato resistance to the postharvest pathogen *Alternaria nees* by activating both the salicylic acid- and jasmonic acid-mediated defense signaling pathways [46] (Table 2).

#### 2.2.2. The CWPs of *P. oligandrum* Possess the Ability to Induce Plant Resistance

The elicitor activity of *P. oligandrum* CWPs effectively contributes to the induction of defense responses in plants. CWPs exhibit a potent capacity to induce resistance in *Arabidopsis* and tomato plants against the bacterial pathogen *R. solanacearum*, effectively mitigating disease development [31,33,47]. CWPs enhance the resistance of rice against the bacterial pathogen *B. glumae* by elevating the gene expression involved in the jasmonic acid (JA) signaling pathway, thereby bolstering the plant’s defense capabilities against the disease [24]. In addition, CWP remarkably bolsters the defense capacity of potato tubers, amplifying their resistance towards the fungal disease known as black scurf [26]. Together, the aforementioned research reports highlight the importance of Oligandrin and CWPs, derived from *P. oligandrum*, in driving plant resistance induction (Table 2).

#### 2.2.3. *P. oligandrum* MAMPs and RXLR Effector Induce Plant Resistance

As it is widely known, the innate immune system of plants indeed consists of two distinct phases. Firstly, PTI (Pattern-Triggered Immunity), also known as the immunity induced by microbe-associated molecular patterns, serves as the primary line of defense in plants. In simple terms, plants can recognize and counteract invaders through their highly conserved pattern recognition receptors (PRRs). The PTI response primarily addresses a wide range of potential pathogens. Secondly, ETI (Effector-Triggered Immunity) is considered the second line of defense in plants. In this stage, plants are capable of recognizing and combating effector molecules of specific pathogens. This response is typically stronger and more effective, as it can lead to a hypersensitive cell death response (HR) and prevent further spread of the pathogenic microorganisms [39]. Currently, some MAMPs and effectors that can induce plant resistance have been identified from *P. oligandrum* (Table 2).

MAMPs can trigger plant defense responses, and Elicitin is a typical class of MAMPs. The CWPs from *P. oligandrum* can trigger plant defense responses. Further studies have discovered two novel Elicitin-like proteins, POD-1 and POD-2, isolated from *P. oligandrum* CWP. These proteins are capable of inducing defense-related gene expression in both sugar beet and tomato, and also enhance sugar beet’s resistance against the fungus *Aphanomyces cochlioides* [48,49]. *Pythium oligandrum* and its Elicitin-like proteins, known as Oligandrins, have been demonstrated to induce disease resistance across a variety of plants. Further research has unveiled that two variants of Oligandrin, Elicitin-like proteins Oli-D1 and Oli-D2, are capable of inducing immune responses in tobacco and tomato [50]. Moreover, our team has identified a novel Elicitin protein, PoEli8, from *P. oligandrum*, which has the ability to confer resistance to *Ph. capsici* in Solanaceae plants. Further research has revealed that a cell surface receptor-like protein (RLP), REli, is capable of perceiving the immune response of PoEli8 [51].

Necrosis- and ethylene-inducing peptide 1 (Nep1)-like proteins (NLPs) represent another category of MAMPs that are pervasively distributed among both eukaryotic and prokaryotic microorganisms. PyolNLP5 and PyolNLP7, derived from *P. oligandrum*, have significantly reduced disease severity and suppressed the in planta growth of *Ph. capsici* in solanaceous plants, including tobacco (*N. benthamiana*), tomato (*S. lycopersicum*) and pepper (*C. annuum*) [52]. NLPs have been postulated to exert bifunctional effects in plant– microorganism interactions, serving simultaneously as elicitors of immune reactions and as virulence factors akin to toxins [53]. A more in-depth investigation elucidated that the plant disease resistance conferred by PyolNLP5 is independent of its necrosis-inducing capabilities and the generation of reactive oxygen species (ROS) [54]. Taken together, the primary research on MAMPs inducing plant resistance in *P. oligandrum* mainly focuses on two types: Elicitin and NLPs (Table 2).

In the interaction between oomycetes and plants, oomycetes secrete a plethora of intracellular effectors to modulate plant defense responses. Of these, RXLR serves as a paradigmatic example of such intracellular effectors. PyolRXLR32, a novel RXLR effector derived from *P. oligandrum*, has been identified by our studies to trigger cell death and the accumulation of reactive oxygen species in tobacco (*N. benthamiana*). Furthermore, it has been proven to possess the capability to enhance the resistance of *N. benthamiana* against *Ph. capsici* [55]. Our discovery represents evidence for the first identification of RXLR effectors in *P. oligandrum* (Table 2).

### 2.3. P. oligandrum Induces Ferroptosis to Enhance Plant Resistance

Ferroptosis, a recently unearthed modality of cellular demise, is characterized by an assemblage of iron and lipid peroxidation. It is remarkable in that it can be elicited within plant cells as a response to both biotic and abiotic stresses, thus broadening the spectrum of its potential impact in the scientific field [56,57,58]. Our research team has pioneered in discovering the capability of *P. oligandrum* to trigger ferroptosis-like cell death, further demonstrating its potential to suppress the attack of *Ph. sojae* within soybean plants [59]. This is a groundbreaking piece of evidence in the realm of scientific breakthroughs.

## 3. *P. oligandrum* Has the Ability to Suppress and Kill Pathogens Through Its Mycoparasitic Interactions

*Pythium oligandrum* not only mitigates pathogens by inducing plant resistance, but it also suppresses, and potentially kills, pathogens through its potent mycoparasitic functions. Exhibiting formidable mycoparasitic properties, *P. oligandrum* has been demonstrated to entwine its hyphae around those of various oomycetes and fungi, including *Pythium*, *Phytophthora*, *Fusarium*, etc. (Table 3). For example, the hyphae of *P. oligandrum* possess the ability to coil around and penetrate the hyphal structures of *Pythium myriotylum* [7,9], and the volatile organic compounds (VOCs) emitted by *P. oligandrum* significantly contribute to its parasitic interaction as well [60]. *Pythium oligandrum* decreased the mycelial growth of the pathogenic *P. aphanidermatum* through mycoparasitism on plates [6,7]. Additionally, *P. oligandrum* exhibits parasitic behavior towards several oomycete pathogens, including *Phytophthora infestans*, *Phytophthora megasperma*, *Pythium ultimum*, *Pythium debaryanum* and *Aphanomyces laevis* [4,5,61,62,63]. *Pythium oligandrum* also has a mycoparasitic effect on various fungal pathogens. Amongst these, *P. oligandrum* manifests parasitism predominantly on the *Fusarium*, incorporating species such as *F. graminearum*, *F. verticillioides*, and *F. oxysporum* [16,62,64,65]. The Microsporum genus, inclusive of *M. canis* and *M. gypseum*, comes next [66,67]. Besides these, it also exhibits parasitic behaviors towards various fungi including *Trichophyton mentagrophytes*, *Rhizoctonia solani*, *Botrytis cinerea*, *Sclerotinia minor*, and *Verticillium dahliae*, among others [22,26,67,68] (Table 3).

## 4. *P. oligandrum* Possesses the Potential to Stimulate and Augment Plant Growth

The phenomenon of growth enhancement by beneficial microorganisms has been extensively documented and is frequently linked to the production of microbial phytohormones and secondary metabolites. *Pythium oligandrum* improves plant fitness by augmenting the root system with tryptamine, a precursor of the phytohormone auxin [2,69]. In recent years, there have been numerous research reports documenting the ability of *P. oligandrum* to stimulate plant growth. *Pythium oligandrum* VOCs have been noted to enhance the growth of ginger, specifically in aspects such as root volume, the quantity of root tips, cumulative root length, and the mean root diameter [8]. The *P. oligandrum* strains MS15, MS19, and MS31 have been found to significantly augment the shoot length and root length of soybeans [6]. Furthermore, *P. oligandrum* not only encourages growth parameters in tomatoes and eggplants such as plant height, fresh weight, and dry weight [11], but it also enhances attributes of the red clover like tap-root diameter, root branching, stand height, and forage yield [35] (Table 4).

## 5. Conclusions

*Pythium oligandrum* has been increasingly recognized by the scientific community for its dual biocontrol mechanisms: mycoparasitism against fungal pathogens and the induction of systemic resistance in host plants. Furthermore, *P. oligandrum* elevates plant growth through the provision of tryptamine to the root system. Consequently, *P. oligandrum* has the potential to exert multifaceted effects on the plant. We have encapsulated the present-day perspective on molecules, such as Oligandrin, CWPs, MAMPs, and RXLR effectors, that foster plant resistance. However, the scale of extant research on these facets remains constrained. Therefore, a pronounced understanding of the molecular mechanisms underpinning these molecules’ modus operandi is essential to harness the formidable capabilities of *Pythium oligandrum* optimally.

## Figures and Tables

**Table 1:** Summary of *P. oligandrum* as biological control agent.

Pathogen Type	Pathogen Species
Oomycete	*Pythium myriotylum*, *Pythium aphanidermatum*, *Pythium diclinum*, *Pythium dissotocum*, *Pythium ultimum*, *Plasmopara viticola*
Fungus	*Fusarium culmorum*, *Fusarium oxysporum*, *Fusarium graminearum*, *Phaeomoniella chlamydospore*, *Erysiphe necator*, *Aphanomyces cochlioides*, *Verticillium wilt*, *Verticillium dahlia*, *Rhizoctonia solani*, *Botrytis cinerea*, *Sphaerotheca macularis*, *Mycosphaerella fragariae*, *Neofusicoccum parvum*, *Sclerotinia sclerotiorum*
Bacteria	*Ralstonia solanacearum*
Nematode	*Meloidogyne incognita*

**Table 2:** Summary of Oligandrin, CWPs, MAMPs and RXLR effectors of *P. oligandrum*.

CWP/MAMPs Type	Pathogen Type	Pathogen Species
Oligandrin	Fungus	*Oidium neolycopersici*, *Botrytis cinerea*, *Fusarium oxysporum*, *Alternaria nees*
	Oomycete	*Phytophthora parasitica*
CWPs	Bacteria	*Burkholderia glumae*, *Ralstonia solanacearum*
	Fungus	*Rhizoctonia solani*
POD-1, POD-2	Fungus	*Aphanomyces cochlioides*
Oli-D1, Oli-D2	Fungus	*Botrytis cinerea*
PoEli8	Oomycete	*Phytophthora capsici*
PyoINLP5, PyoINLP7	Oomycete	*Phytophthora capsici*
PyoRXLR32	Oomycete	*Phytophthora capsici*

**Table 3:** Summary of mycoparasite of *P. oligandrum*.

Pathogen Type	Pathogen Species
Oomycete	*Pythium myriotylum*, *Pythium aphanidermatum*, *Phytophthora infestans*, *Pythium ultimum*, *Phytophthora megasperma*, *Pythium debaryanum*, *Aphanomyces laevis*
Fungus	*Fusarium graminearum*, *Fusarium verticillioides*, *Fusarium oxysporum*, *Microsporum canis*, *Microsporum gypseum*, *Trichophyton mentagrophytes*, *Rhizoctonia solani*, *Botrytis cinerea*, *Sclerotinia minor*, *Verticillium dahlia*

**Table 4:** *P. oligandrum* promotes plant growth.

Plant Species	Growth-Promoting Characteristics
Ginger (*Zingiber officinale* Roscoe)	Root volume, number of root tips, total root length, and average root diameter
Soybean (*Glycine max* L.)	Shoot length, Root length
Tomato (*Solanum lycopersicum* L.), Eggplant (*Solanum melongena* L.)	Plant height, Fresh weight, Dry weight
Red clover (*Trifolium pratense* L.)	Tap-root diameter, Root branching, Stand height, Forage yield

## Data Availability

No new data were created or analyzed in this study. Data sharing is not applicable to this article.
